# Design of MIR Dispersive Spectrograph System with Uncooled Microbolometer

**DOI:** 10.3390/s23042205

**Published:** 2023-02-15

**Authors:** Pattarapong Sunongbua, Suwan Aekram, Weerasak Lertsiriyothin

**Affiliations:** School of Agricultural Engineering, Institute of Engineering, Suranaree University of Technology, Nakhon Ratchasima 30000, Thailand

**Keywords:** mid-infrared spectrograph, diffraction grating, microbolometer

## Abstract

To make the mid-infrared (MIR) dispersive spectrograph a practical tool in industrial food processing lines, we designed a dispersive spectrograph system with an uncooled microbolometer focal plane array (FPA) detector for MIR spectral acquisition. To precisely regulate the angle of a rotatable grating to acquire the MIR spectrum, the spectral resolution and spatial resolution of the system were rigorously controlled to improve system performance. In the reflectance operation mode of the MIR dispersive spectrograph, the uncooled microbolometer FPA detector offered a maximum spectral resolution of 12 nm for the MIR, when a 300 grooves/mm blazed grating was used. Utilizing an optical parametric oscillator (OPO) pulse laser source, the wavelengths of the first-order diffraction were validated, and the system’s spectral resolution limit was determined. As a line-scanning source, a Globar broadband source was installed, and the USAF 1951 Resolution Calculator was used to establish the spatial resolution of the imaging spectrograph. Using NI LabView, the logical operational technique for controlling the MIR dispersive spectrograph was encoded into system firmware. The GUI and test results are thoroughly described.

## 1. Introduction

Infrared spectroscopy is frequently used to evaluate agricultural and food products, including in polysaccharide structure studies [1] and quality determinations of various liquid-based foods [2]. Despite the fact that mid-infrared (MIR) spectroscopy has been proven to accurately predict the adulteration of numerous foods, such as honey [3,4], and been used to monitor chemical reactions in food processing [5], the technique is still not widely used. Due to the limitations of a standard IR spectrometer, almost all testing is conducted within a micro-spot.

For the practical application of MIR spectroscopy in food processing, such as the inspection of processed foods and agricultural products [6], line scanning is a fundamental system requirement. Typically, the array, or line scanning, spectrograph requires a dispersive principle to separate the wavelengths of a broadband light source.

When a wavelength separates the infrared spectrum with a dispersive spectrograph, the diffraction grating must be rotated accurately to resolve the wavelength at the nanometer level while covering a broad MIR spectrum. As a grating function, the diffraction grating rotates in a wavelength-dependent pattern [7,8]. To obtain the complete MIR spectral range, an array dispersive spectrograph requires a comparatively large focal plane array (FPA) detector, in contrast to the visible spectral range. To capture the notoriously weak MIR absorbance or transmittance intensity, not only is a relatively large FPA required, but so is a detector with a high sensitivity. In general, photovoltaic liquid N_2_-cooled detectors are composed of mercury cadmium telluride (MCT) [9,10], indium antimonide (InSb) [11], or deuterated lanthanum alanine-doped triglycine sulfate (DLaTGS) as a single point detector fitted in an infrared spectrometer. The practical use of an MIR spectrograph in a food processing line is impeded by the high cost of an array of liquid N_2_-cooled detectors fabricated from these materials. Due to its lower sensitivity, longer response time, and lower signal-to-noise ratio (SNR), the uncooled microbolometer detector has not been routinely utilized in spectrometers or spectrographs. The uncooled microbolometer, on the other hand, does not require liquid N_2_ cooling to maintain minimal dark noise and great signal stability, has no requirement for rare elements, and has a significantly lower price than liquid N_2_-cooled detectors.

Previously, microbolometers have been utilized in infrared spectrometers [12,13]. The microbolometer FPA is used for spectrograph imaging. Novel types of microbolometer FPA detectors, with relatively small pixel sizes, low Noise Equivalent Temperature Difference (NETD), and new materials have boosted the performance of essential characteristics [14,15]. These qualities offer considerable enhancements to the spectral resolution, sensitivity, and wavelength range of the spectrograph. Such spectral and spatial acquisition systems with good characteristic requirements and reasonable measurement times are currently on the market. For the infrared spectrograph system, the microbolometer is an alternative detector to the MCT and InSb detectors. To specify the limit factors for both spectral and spatial resolution, however, rigorous evidence for the successful use of an MIR dispersive spectrograph with a microbolometer is still necessary.

For the design of a bespoke system, employing optomechanical technologies, an optical system was controlled by an NI LabView virtual instrument system. Controlling the revolving grating necessitated the creation of an NI LabView-controlled virtual instrument [16,17]. Using the virtual equipment, for instance, the Laser-Induced Fluorescence Spectroscopy system [18] and the High-Performance Liquid Chromatography Lab [19] were successfully controlled.

The purpose of this study is to construct an MIR dispersive spectrograph equipped with a microbolometer FPA detector to identify the key constraints of spectrum acquisition, namely, spectral resolution, spatial resolution, and operating speed. These parameters are incredibly useful for developing firmware for a high-resolution and dependable dispersive spectrograph with a microbolometer detector. Here, the microbolometer FPA (35 μm pixel size) is utilized as a detector of the dispersive spectrograph, yielding different wavelengths of the first-order diffraction, and spatial positions distinguishable from the array data. The MIR dispersive spectrograph system’s measurement time is shortened. The signal resolution of the MIR dispersive spectrograph system is thereby improved. An optical parametric oscillator (OPO) pulse laser source was utilized to confirm unique wavelengths diffracted at any angle and to define a signal for a diffracted wavelength. The upper limit of the spectral resolution within the MIR spectrum was firmly determined. In addition, the spatial resolution of the spectrograph was determined using the USAF 1951 Resolution Calculator (Edmund Scientific). To confirm the correctness of the diffraction wavelength and optical resolution, a line scan test was conducted using an IR standard polystyrene film.

## 2. Configuration of the System

### 2.1. Optical Design

The Czerny–Turner spectrograph inspired the development of an MIR dispersive spectrograph system. When light enters the entrance slit, the amount of light reflected from the sample is determined. Collimation of the reflection light is performed to collect both specular and diffuse reflectance light from the non-shining organic sample to enhance the energy beam entering the spectrograph, where the beam is collimated with an off-axis parabolic (OAP) mirror before being reflected into the diffraction grating.

**Figure 1:**
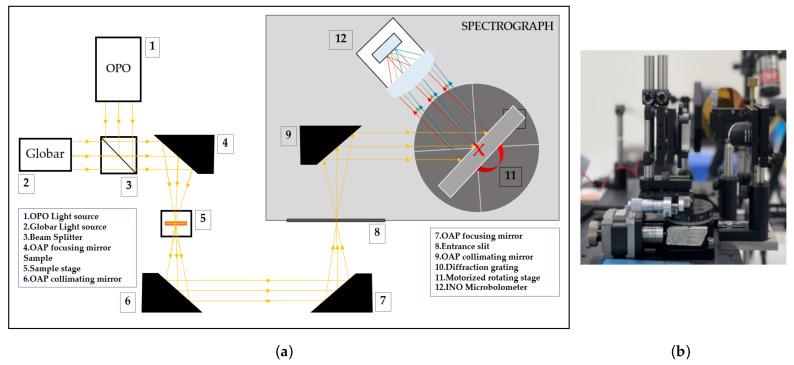
(**a**) The mid-infrared (MIR) dispersive spectrograph system. (**b**) The rotating diffraction grating with a motorized rotating stage system.

Figure 1a depicts the MIR dispersive spectrograph system’s design. An illumination system, composed of a stabilized globar light source (500 to 9000 nm, Thorlabs, Newton, NJ, USA) and OPO pulse laser source (NT200 Series, Ekspla, Vilnius, Lithuania), emits in the MIR spectral range through OAP4 to focus a beam onto the sample. The reflection light from the sample is collected by OAP6 and focused by OAP7 into the spectrograph’s entrance slit. The MIR dispersive spectrograph is composed of an OAP mirror, a diffraction grating, and an uncooled vanadium oxide (VOx) microbolometer FPA detector (3–14 μm) with a focusing lens (INO, Microxcam 384).

In the optical wavelength separation system, the MIR wavelength is diffracted using a reflection grating. The common groove surface of the MIR reflection grating has 150 to 300 grooves per millimeter and is covered with highly reflective aluminum. The diffraction wavelength depends on the incidence and diffracted angles of light from the grating facet, as described by the grating function, Equation (1), where d is the blazing spacing, α is the incidence angle of the light relative to the grating normal, and β is the diffraction angle of the light at the m^th^-order of diffraction light.

(1) mλ=d(sinα+sinβ)

As illustrated in Figure 1b, a rotating diffraction grating system consists of (1) a motorized rotating stage with a stepper motor, (2) a linear stage, and (3) a diffraction grating (300 grooves/mm, GR2550-30035, Thorlabs) with a mounting plate. Mounting a diffraction grating on a linear stage, with the center of the grating surface aligned with the rotation center of the motorized rotating stage, stabilizes the incidence and reflected angles as the grating is revolved. A microbolometer FPA detector with an infrared focusing lens collects the dispersive spectral signal. Utilizing a focusing lens with a high infrared transmission rate maximizes the low intensity of the MIR energy and focuses the collimated beam onto the micropixel of the microbolometer detector. The motorized rotating stage is electronically controlled by a stepping motor controller to provide the necessary rotation for measurement. NI LabView is used to program the system’s operational software to regulate and acquire the infrared spectral signals.

### 2.2. Programming Method

**Figure 2:**
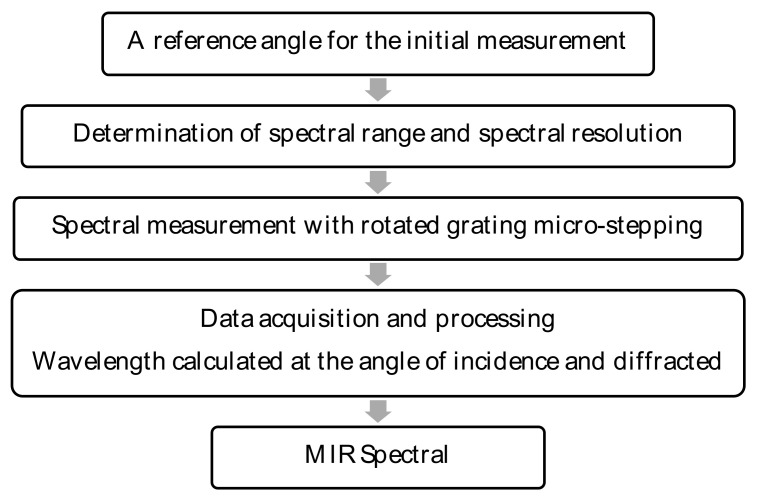
The schematic diagram for controlling the MIR dispersive spectrograph.

**Figure 3:**
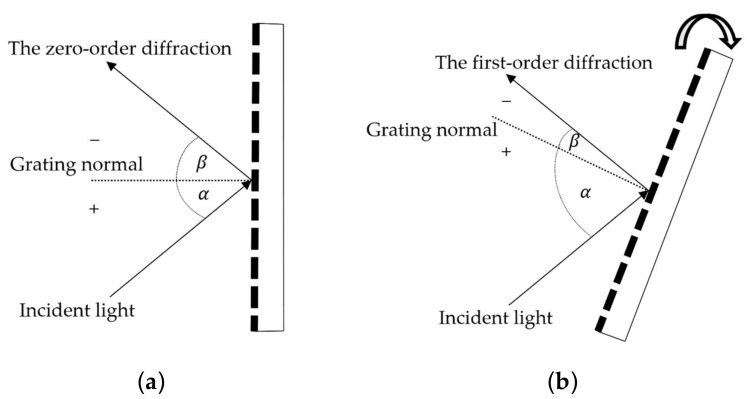
The reflection angle of (**a**) the zero-order diffraction and (**b**) the first-order diffraction with rotated grating.

Figure 2 depicts the algorithm for controlling the MIR dispersive spectrograph system to measure spectral wavelength signals. As shown in Figure 3, the zero-order diffraction angle (α = −β) position was first tuned as a reference angle for the clockwise rotation of the diffraction grating to a specified angle, corresponding to the predetermined first-order diffraction wavelength. However, the rotation angle continues to be constrained by a parallel line between the incident light and the grating facet.

To acquire a precise wavelength resolution (e.g., 100 nm), it is crucial to determine the rotation angle step of the diffraction grating; consequently, a stable rotating angle of the diffraction grating is essential. Using the diffraction grating equation and the reference position of the zero-order diffraction, as shown in Equation (2), the rotation angle of the diffraction grating is calculated. The rotation angle of a diffraction grating is computed based on the difference between the target wavelength (*λ*_n_) at the *m_n_*-order diffraction and the wavelength (*λ*_0_) at the zero-order diffraction (*m*_0_ = 0).

(2) $$m_{n}\lambda_{n}-m_{0}\lambda_{0}=d(\sin\alpha_{n}+\sin\beta_{n})-d(\sin\alpha_{0}+\sin\beta_{0})\;\;$$

In the case of the first-order diffraction (*m_n_* = 1), the target wavelength equation can be rearranged mathematically as follows:

(3) $$\frac{\lambda_{n}}{d}=2\cos(\frac{\alpha_{n}+\alpha_{0}}{2})\sin(\frac{\alpha_{n}-\alpha_{0}}{2})+2\cos(\frac{\beta_{n}+\beta_{0}}{2})\sin(\frac{\beta_{n}-\beta_{0}}{2})\;$$

When a microbolometer FPA is fixed in place and a diffraction grating is rotated, the incident and diffraction angle differences for the zero- and first-order diffractions are equal. As a result, the incident angle of the first-order diffraction can be replaced with that of the reference zero-order diffraction, as shown below:

(4) $$\alpha_{1}-\beta_{1}=\alpha_{0}-\beta_{0}\;$$

(5) $$\alpha_{1}=\alpha_{0}+\sin^{-1}\left(\frac{\lambda_{1}}{2d\cos\alpha_{0}}\right)$$

Equation (5) was utilized to determine the precise incident angle required to achieve any desired spectral wavelength This equation was incorporated into the algorithm for controlling the grating position in order to achieve any user-specified diffraction wavelength.

### 2.3. Program for Rotating Motorized Stage

The diffraction grating is mounted on a motorized rotating stage with a 1:90 motor ratio and a step angle of 1.8 degrees per step, which is controlled by a pulse signal (in one period, one pulse causes the stepping motor to rotate by one step). We use the Arduino board and the micro-stepper driver to control the rotation angle of the motorized rotating stage, by adjusting the frequency and duration of the pulse signal.

**Figure 4:**
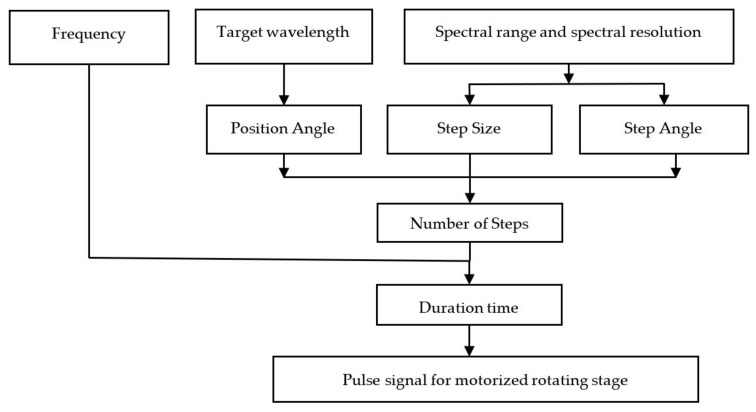
The flowchart diagram for controlling the stepper motor of the motorized rotating stage.

Figure 4 illustrates the algorithm for calculating the pulse signal used to control the rotation of the motorized rotating stage. The wavelength resolution limit is used to calculate the step size and angle of the motorized stage, whereas the wavelength of interest specifies the position angle. The number of pulse signal steps is computed on the basis of the position angle, step size, and step angle from Equation (6).

(6) $$\mathrm{Number}\;\mathrm{of}\;\mathrm{Steps}=\frac{\mathrm{Position}\;\mathrm{Angle}}{\mathrm{Step}\;\mathrm{Angle}\;\times \;\mathrm{Step}\;\mathrm{Size}}$$

where the Number of Steps is the number of pulses in the pulse signal, Position Angle is the desired angle of rotation for the stepper motor in degrees, Step Angle is the angle of rotation of the stepper motor in one pulse [Full Step = 1.8 degree/pulse], and Step Size is the resolution of rotation in each pulse [Full step=1]. For the duration of the pulse signal, the frequency and number of steps for controlling the rotation of the diffraction grating were then determined.

### 2.4. Data Acquisition and Processing

**Figure 5:**
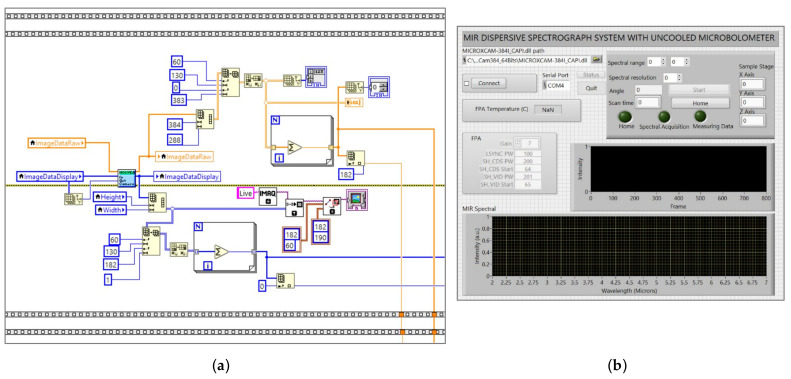
(**a**) NI LabView VI system for mapping an MIR signal from a microbolometer focal plane array (FPA) detector (**b**) Graphical user interface for MIR dispersive spectrograph.

The diffraction signal from a reflection grating was detected by a focusing lens-equipped microbolometer FPA detector. A Gigabit Ethernet Link was utilized for communication between the microbolometer FPA detector and a computer. Figure 5a depicts the LabView VI system. The block diagram is utilized to acquire an image from the microbolometer FPA. It provides a 16-bit intensity raw signal for a pixel. The IMAQ was used to acquire the signal and display the spectral signal on the graphical user interface. The wavelength was determined by applying Equation (5) to the formula node sub VI, where the input of the formula node is a rotating angle of the diffraction grating (angle of incidence and diffraction). Then, we used a for-loop to correlate a change in the rotation angle of the diffraction grating with the corresponding wavelength (each incremental counter of the for-loop is equivalent to one rotating step), ensuring that precise mapping of each pixel with the correct MIR spectral wavelength was acquired.

As illustrated in Figure 5b, an operation of the MIR dispersive spectrograph can be carried out via a firmware interface with the LabView GUI program. Before performing any measurement, firstly, the user presses the home button to find the zero-order diffraction position. The system will automatically locate the maximum intensity to determine the zero-order diffraction position. Users can also adjust the resolution of the rotation diffraction grating to measure the signal. A real-time spectral signal that maps the signal to its wavelength is depicted in the graph. Starting at the reference rotation angle (the zero-order diffraction), the wavelength is determined based on the angle of the incident and diffracted light. The infrared spectral data can be saved as a .txt file or an .xls file, with the spectral intensity of the wavelength (y-axis), plotted versus wavelength, incidence angle, and diffracted angle (x-axis).

## 3. Results and Discussion

### 3.1. Spectral Characteristics

**Figure 6:**
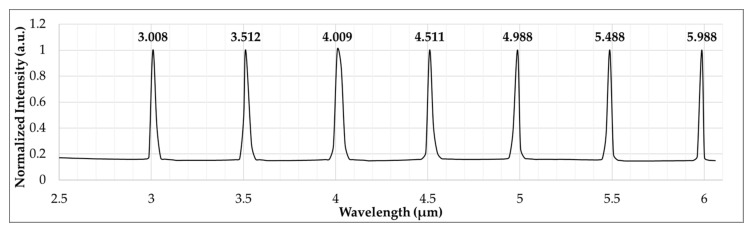
Wavelength validation of the first-order diffraction spectral acquired by the MIR dispersive spectrograph with an optical parametric oscillator (OPO) pulse laser source for the wavelength range of 3 to 6 μm.

The MIR dispersive spectrograph is constructed according to the Czerny–Turner principle [20,21], and an uncooled microbolometer FPA detector is utilized to detect dispersive infrared spectral signals, with rotating gratings at angles corresponding to Equation (5). The optical alignment of the MIR dispersive spectrograph produces the reference position of the zero-order diffraction at an incident angle of 14.5 degrees and a diffraction angle of −14.5 degrees. To validate the spectral diffraction signals of the system, the diffraction wavelength validation method employs a tuneable OPO pulse laser source. As shown in Figure 6, the first-order diffraction signals from a ruled reflective diffraction grating (design wavelength of 3.5 μm) were acquired from 3 to 6 μm, with an incremental wavelength of 0.5 μm. Compared to the OPO laser, the MIR dispersive spectrograph provided a wavelength of the first-order diffraction infrared signal with a maximum error of 0.007 percent. 

Since the MIR wavelength is a long wavelength region, overlapping spectra between the first-order and second-order diffraction are nearly impossible for a ruled grating with a 26.5-degree blaze angle. With a relatively intense OPO pulse laser, the second-order diffraction of 3 μm appeared on the same pixel as the first-order diffraction of 6 μm, but with much less intensity. This suggests that the MIR dispersive spectrograph, when equipped with a typical broadband globar light source, generates a signal of negligible second-order diffraction. Therefore, it offers a free spectral range within the wavelength range specified by the grating.

**Figure 7:**
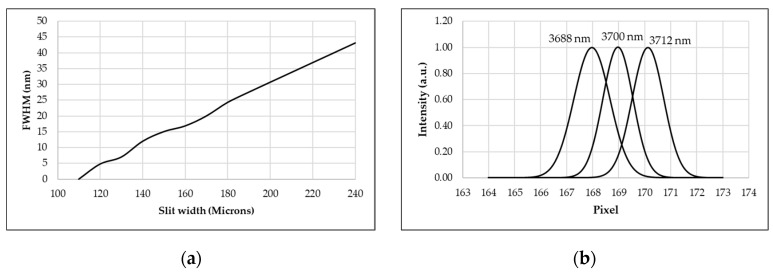
(**a**) The relationship between the FWHM and the width of the entrance slit (**b**) Spectral resolution of the system with a central wavelength of 3700 nm.

Figure 7 depicts the diffraction signal characteristic of the MIR dispersive spectrograph system. The full width at half-maximum (FWHM) of the diffraction signal has a substantial influence on the spectral resolution of any dispersive spectrograph [21]. Figure 7a demonstrates that the FWHM of the first-order diffraction signal acquired by our system is 30 nm for a slit width of 200 µm. Positive linearity was observed in the FWHM derivative as a function of slit width, resulting in a reduction in the system’s spectral resolution for wider slit widths.

By varying the wavelength of the OPO laser around its central wavelength, the spectral resolution of the system was analyzed. Since each wavelength’s energy falls on distinct pixels of the microbolometer FPA, the closest separable spectral wavelength can be identified. As shown in Figure 7b, the central wavelength was set to 3700 nm, and the OPO laser wavelength was adjusted by 1 nanometer around the central wavelength. The system’s resolution is 12 nm/pixel at 3700 nm with a slit width of 140 μm due to the resolving spectra. For a grating with 300 grooves/mm, the system’s angular dispersion is 107.43 nm/degree. Notably, an MIR dispersive spectrograph with a microbolometer FPA should not have an excessively narrow slit, as the weak energy intensity of a typical globar broadband light source would not be detectable, and the system’s sensitivity would be significantly diminished.

Even though an MIR diffraction grating mounted on a rotating motorized stage with a micro stepper motor can be tuned to a precise angle, the MIR diffraction grating’s resolving power limits the fine rotational step angle of a diffraction grating intended to enhance spectral resolution. With a maximum spectral resolution of 12 nm, the system requires 4.5 s to acquire 1 μm of the mid-infrared spectrum over a sample length of 10 mm. The maximum spectral resolution of our system is comparable to that of other MIR dispersive spectrometers equipped with MCT detectors, whose spectral resolutions range from 40 to 65 nm, with slit widths ranging from 108 to 153 μm [9].

According to the free spectral range and the spectral resolution, the MIR dispersive spectrograph can collect the MIR signal in the wavelength range of 3 to 6 μm with a single diffraction grating. To cover the full MIR spectrum and improve the spectral resolution, the installation of a double diffraction grating with other grating blaze angles or groove/mm would fulfill the requirements [22].

**Figure 8:**
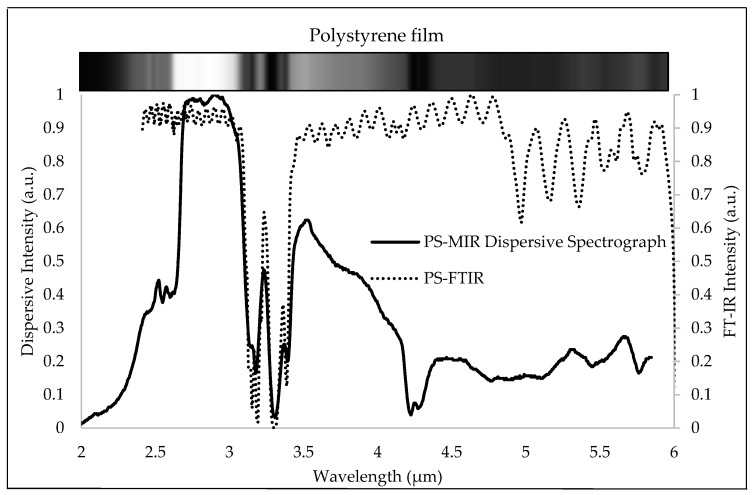
The transmittance spectral profiles of the MIR-certified wavelength standard polystyrene film acquired by the MIR dispersive spectrograph and FTIR.

The reliability of the MIR spectral signal acquired by the MIR dispersive spectrograph system was proved by measuring spectrometer calibration polystyrene film (Mid Infrared certified wavelength standard SN:2809, Brucker Optics) [9,10,11] and switching to a globar light source, following spectral validation with an OPO pulse laser source. As the primary illumination source, a globar light source was installed in the MIR dispersive spectrograph. Figure 8 depicts the MIR spectral profile (2–6 µm) of the standard polystyrene (PS) film, measured at 20 °C room temperature. The major absorption peaks of PS were between 3 and 3.5 µm, which corresponded well with the location of its standard MIR spectral profile as measured by an FTIR spectrometer with a resolution of 8 cm^−1^. The limitation of the microbolometer with a focusing lens, which the manufacturer claims detects well at wavelengths above 3 µm, and the grating efficiency of “3.5 µm Design Wavelength Reflective Diffraction Gratings” led to a low intensity in the wavelength range of 2 to about 2.7 µm. At the same time, the low intensity for wavelengths greater than 4.5 µm pertained to the Spectral Power of the SLS203L Stabilized Globar Light Source. In addition, the effect of CO_2_ gas in ambient air was perceivable at wavelengths close to 4.2 µm [23]. Moreover, when a Globar light source oriented along the y-axis was employed, the spatial signal of the sample was of high spectral quality.

### 3.2. Spatial Resolution

**Figure 9:**
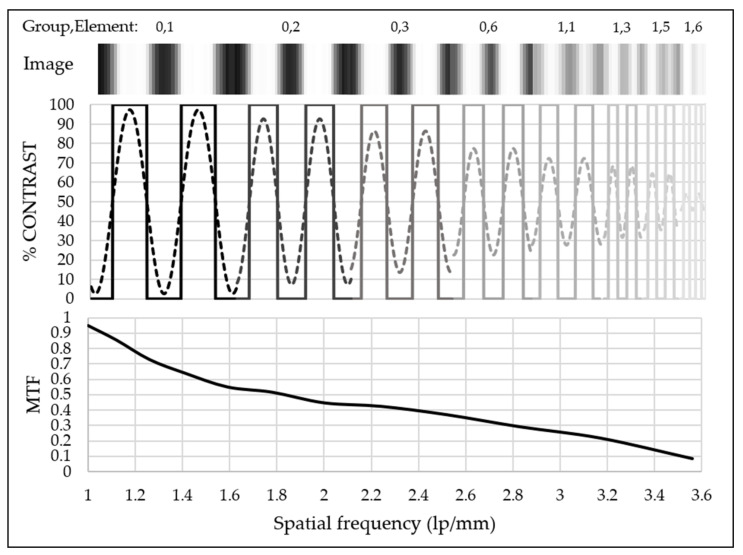
The image of USAF 1951 Resolution Calculator group 0 and 1 with elements 1 to 6 on the y-axis pixel of the microbolometer FPA with the corresponding % contrast of both a bar target pattern (-) and image pattern (--) and the spatial resolution of the spectrograph by the modulation transfer function (MTF) with the spatial frequency.

A microbolometer FPA installation in an MIR dispersive spectrograph enables the measurement of signals from the x-axis and y-axis pixels of the FPA, thereby enabling 2D imaging of the sample. The horizontal pixel (x-axis) identifies wavelength, whereas the vertical pixel (y-axis) designates line-spatial. The USAF 1951 Resolution Calculator was utilized to calibrate the line-spatial resolution limit [24]. Signals from three vertical elements are compared to determine the spatial configuration of the y-axis. As shown in Figure 9, the width and spacing of each vertical element signal were in accordance.

**Figure 10:**
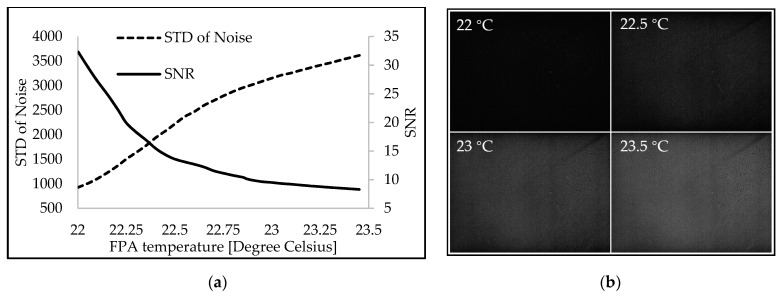
Effect of the FPA temperature of the microbolometer FPA operating at room temperature on (**a**) the standard deviation of the background noise and the signal-to-noise ratio (SNR). (**b**) Images of the background noise at three operating temperatures.

At a spatial frequency of less than two line pairs per millimeter (lp/mm), the modulation transfer function (MTF), or contrast clear separation, was experimentally demonstrated using group numbers zero to one and elements one to six of the USAF. The MTF values indicated that the system has a spatial resolution limit of 3.5 lp/mm, which is equivalent to 140 μm of the element; consequently, this parameter was determined for an incremental step size for a 2D scan of the array imaging spectrograph. Figure 10 is an example of a 2D image acquired by the MIR dispersive spectrograph of the USAF 1951. According to the 300 mm working distance of the MIR focusing lens, the FPA microbolometer cannot be positioned any closer to the diffraction grating. The spatial resolution is subsequently constrained because the detector cannot be moved closer, to increase the MTF.

### 3.3. Signal-To-Noise Ratio

Since the microbolometer FPA utilized here is an uncooled thermal detector, vanadium oxide was used to create it [14,15]. During continuous measurement of the spectral signal for a slow rotating speed of the diffraction grating, it is necessary to account for the heat-sensitive nature of the thermal detector, which contributes to the rise in the FPA’s temperature. To determine the actual performance of the detector, the SNR of the first-order diffraction signal was calculated using the SNR value or contrast-to-noise ratio, as shown in Equation (7) [25,26].

(7) $$\mathrm{SNR}=\frac{\left|\mu_{s}-\mu_{N}\right|}{\sigma_{N}}$$

The SNR is the absolute value of the difference between the average signal intensity ($\mu_{s}$) and the background noise ($\mu_{N}$) divided by the standard deviation of the background noise ($\sigma_{N}$). With an entrance slit width of 140 μm and an integration time of 20 milliseconds, the MIR dispersive spectrograph was validated. Figure 10a demonstrates that the standard deviation in the background noise recorded by the microbolometer FPA detector increased nonlinearly with a small change in the FPA’s temperature (approximately 1.5 degrees Celsius), while the SNR of the system rapidly decreased. Figure 10b reveals, however, that the background image of the noise signal was undetectable when the FPA temperature was brought down to 22 °C or lower.

In addition, it is simple to maintain a relatively constant temperature of the system’s uncooled microbolometer, by operating it in a room cooled by air conditioning, to obtain a signal with high spectral quality.

The significant parameters of the MIR dispersive spectrograph are summarized in Table 1.

## 4. Conclusions

In this paper, an MIR dispersive spectrograph system, equipped with an uncooled microbolometer FPA detector, was developed to evaluate all of the parameters that play a significant role in the MIR spectral acquisition. The system was constructed and tested, and found to function effectively with non-specular reflectance samples. The spatial resolution was limited to 3.5 lp/mm, or 140 μm, while the spectral resolution was confined to 12 nm/pixel. With a double grating turret installation, the MIR dispersive spectrograph can acquire the full spectrum of the MIR. The MIR thermal detector platform operating at 20 °C room temperature has acceptable SNR values for use in the MIR dispersive spectrograph. This detector core also supports line spatial, allowing it to be utilized in MIR line-scanning applications. For user-friendly operation, a LabView-based graphical user interface, that considers all parameter limits, was developed as system firmware. Adjustable parameters include the spectral range, spectral resolution, and region of interest.

## Figures and Tables

**Table 1:** Compilation of parameters of the MIR dispersive spectrograph.

Parameters	Values
Spectral range	2–6 μm
Disperser	300 grooves/mm grating
Detector type	Uncooled microbolometer VOx
Detector size	35 μm pixel pitch
Spectral resolution	12 nm/pixel at 3700 nm
Scan time	4.5 s/μm for 10 mm spatial length
Spatial resolution	3.5 lp/mm or 140 μm
Signal-to-noise ratio	32 (SNR, operated at 20 °C)

## Data Availability

The data presented in this study are available on request from the corresponding author.
